# Antimicrobially Active Semen Extenders Allow the Reduction of Antibiotic Use in Pig Insemination

**DOI:** 10.3390/antibiotics10111319

**Published:** 2021-10-29

**Authors:** Anne-Marie Luther, Thu Quynh Nguyen, Jutta Verspohl, Dagmar Waberski

**Affiliations:** 1 Unit for Reproductive Medicine, Clinic for Pigs and Small Ruminants, University of Veterinary Medicine Hannover, Foundation, 30559 Hannover, Germany; anne-marie.luther@tiho-hannover.de (A.-M.L.); thu.quynh.nguyen@tiho-hannover.de (T.Q.N.); 2 Institute for Microbiology, University of Veterinary Medicine Hannover, Foundation, 30173 Hannover, Germany; jutta.verspohl@tiho-hannover.de

**Keywords:** boar, spermatozoa, semen preservation, antibiotics, semen extender

## Abstract

Antibiotic use in semen extenders for livestock may contribute to the development and spreading of multi-drug resistance. Antimicrobial control in semen doses for artificial insemination of pigs is indispensable due to the relatively high storage temperature (17 °C). The objectives of this study were first, to examine whether the antimicrobial capacity differs between antibiotic-free extenders and second, to determine whether an antimicrobial active extender provides the possibility to reduce antibiotics. Antibiotic-free semen extenders Beltsville Thawing Solution (BTS) and Androstar Premium were inoculated at 10^3^ to 10^4^ CFU/mL with four pure bacterial strains isolated from boar ejaculates or a mixture thereof, and then stored for 144 h at 17 °C. Bacterial counts after aerobic culture decreased in BTS up to one log level and decreased in Androstar Premium by 2 to 3.5 log levels (*p* < 0.05). In semen samples from nine boars stored in the inoculated Androstar Premium extender containing half of the standard concentration of gentamicin, bacteria counts were below 10^1^ CFU/mL. Likewise, half of the standard dose of apramycin and ampicillin was fully antimicrobially active and sperm quality was maintained. In conclusion, semen extenders with intrinsic antimicrobial activity allow a reduction in antibiotic use in pig insemination.

## 1. Introduction

Adding antibiotics to semen extenders is a standard procedure in artificial insemination (AI) of livestock to avoid bacterial growth in semen doses during the preservation period. The necessity for antimicrobial control results from the unavoidable presence of bacteria in raw semen naturally stemming from the male and, to a different extent, from the environment. With adaptation of the one-health concept, researchers and the animal breeding industry are seeking strategies to reduce or replace antibiotics in semen extenders. This particularly applies to artificial insemination in pigs as this is the most common breeding technology used in more than 90% of the sows in the main pork-producing countries [1]. The special challenge in pig breeding results from the relatively high semen storage temperature (15–18 °C) favouring bacterial growth, the high volume of insemination doses (50 to 100 mL, two to three semen doses per cycle), and the environmental pollution due to seminal backflow into manure (approximately 85% of the inseminated volume; [2,3]), thus providing a source and reservoir of resistance genes in soil and water [4,5]. Attempts to replace conventional antibiotics for boar semen preservation range from the use of alternative antimicrobial substances such as peptides [6,7,8,9], phytoextracts [10,11] or nanoparticles [12], to the physical removal of bacteria through single layer centrifugation [13,14] and microfiltration [15]. However, until now, none of the concepts have been feasible for AI practice because they lack antimicrobial efficiency, are sperm-damaging or impracticable and costly. The recently proposed antibiotic-free semen storage at 5 °C to achieve bacteriostasis was successfully tested in field insemination trials [16,17] but awaits further proof of concept in AI practice. Therefore, fast realisable alternatives which at least reduce the use of antibiotics would mitigate the current situation until other options become available. 

This led us to study the intrinsic antimicrobial effectivity of two types of semen extenders. Typically, boar semen extenders are based on glucose-salt solutions with a bicarbonate buffer system, like the traditional Beltsville Thawing Solution (BTS [18]), which is still the most used extender in many European countries for short-term preservation up to 4 d [19]. Based on this composition, a series of extenders have come on the market, mostly spiked with sperm membrane protecting agents and additional powerful buffers to achieve long-term preservability up to 7 d [20]. Basic semen extender components, like EDTA and bicarbonate, are known to be antimicrobially effective, alone or in synergism with antimicrobial agents [21,22]. Although extender ingredients are no longer published, it is reasonable to hypothesise that antibiotic-free semen extenders differ in their intrinsic antimicrobial capacity. If verified, the second hypothesis then is that extenders with a higher effectivity against bacteria allow the current standard concentration of antibiotics to be reduced without compromising antimicrobial efficiency and sperm quality. To this end, a series of experiments testing the antimicrobial capacity and the effect on boar sperm quality of two types of semen extender was performed with standard and reduced concentration of conventional antibiotics.

## 2. Results

### 2.1. Experiment 1: Comparison of Bacterial Growth in Nutrient Broth and Antibiotic-Free BTS Extender

**Figure 1:**
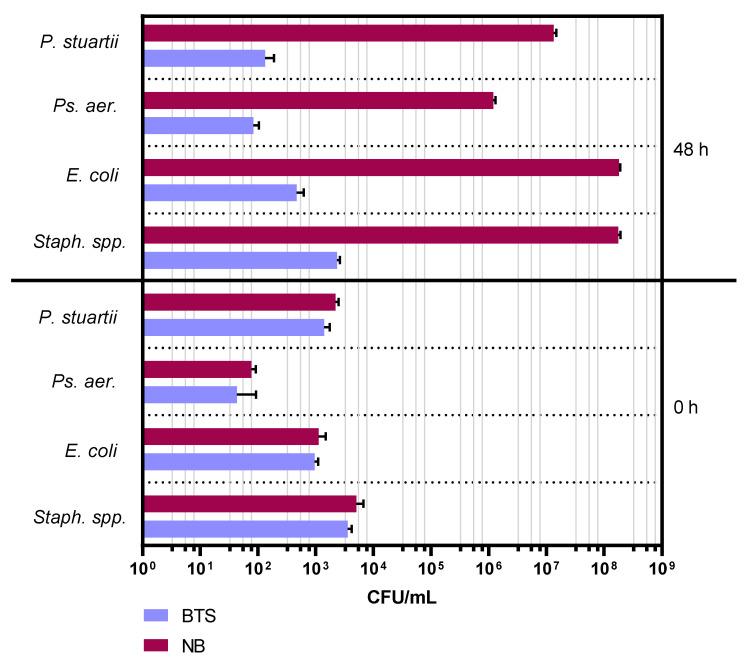
Bacterial counts (CFU/mL) in antibiotic-free semen extender Beltsville Thawing Solution (BTS) and nutrient broth (NB) after inoculation with pure bacterial strains (0 h) and after 48 h of storage at 17 °C. Inoculated bacterial strains were: *P. stuartii*, *Ps. aer., E. coli* and *Staph.* spp. Data are means and SD of three replicates (*n* = 3, Experiment 1).

In BTS extender, the inoculated bacteria reproduced less compared to a conventional nutrient broth (Figure 1). After 48 h of storage at 17 °C, there was almost no bacterial growth in BTS (*Staphylococcus species* (*Staph.* spp.): 2.3 × 10^3^ ± 3.1 × 10^2^; *Escherichia coli (E. coli)*: 4.7 × 10^2^ ± 1.5 × 10^2^; *Pseudomonas aeruginosa* (*Ps. aer.*): 8.3 × 10^1^ ± 2.1 × 10^1^; *Providencia stuartii (P. stuartii)*: 1.3 × 10^2^ ± 5.8 × 10^1^ CFU/mL). After storage at 17 °C in nutrient broth, bacterial counts increased dramatically between 3 and 8 log levels (*Staph.* spp.: 1.8 × 10^8^ ± 1.5 × 10^7^; *E. coli*: 1.8 × 10^8^ ± 1.0 × 10^7^; *Ps. aer.*: 1.2 × 10^6^ ± 1.0 × 10^5^; *P. stuartii*: 1.3 × 10^7^ ± 1.5 × 10^6^ CFU/mL).

### 2.2. Experiment 2: Comparison of Bacterial Growth in Two Different Types of Antibiotic-Free Extenders 

**Figure 2:**
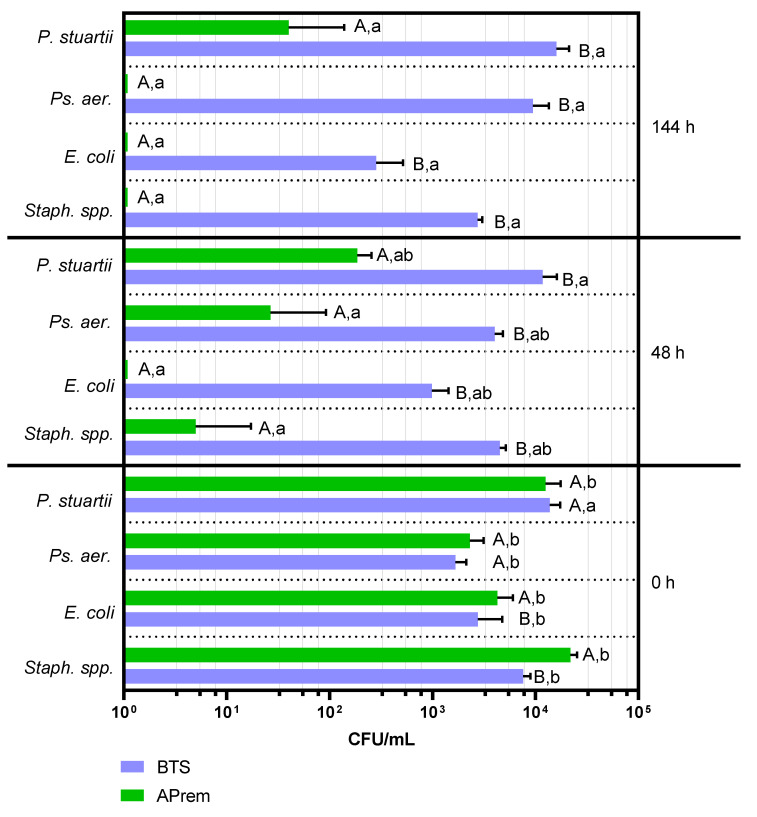
Bacterial counts (CFU/mL) in antibiotic-free semen extenders Beltsville Thawing Solution (BTS) and Androstar Premium (APrem) after inoculation with pure bacterial strains (0 h) and after storage at 17 °C. Inoculated bacterial strains were: *P. stuartii*, *Ps. aer.*, *E. coli* and *Staph.* spp. Data are means and SD (*n* = 6, Experiment 2). A, B: Different capital letters indicate differences between extenders within storage time point (*p* < 0.05). a, b: Different lowercase letters indicate differences between storage time points within extender (*p* < 0.05).

The semen extender and the storage duration at 17 °C had a significant effect on bacterial counts (*p* < 0.05) (Figure 2). At 48 h, bacterial counts in Androstar Premium samples were lower compared to 0 h and compared to BTS at the same time point. After 144 h of storage, bacteria counts in Androstar Premium samples were reduced below the detection limit for *Staph.* spp., *E. coli, Ps. aer.,* and the bacterial growth of *P. stuartii* was significantly lower (4.0 × 10^1^ ± 9.8 × 10^1^ CFU/mL) compared to BTS samples (1.6 × 10^4^ ± 5.2 × 10^3^ CFU/mL). In BTS samples, bacterial counts for *Staph.* spp. *E. coli, Ps. aer.* decreased by up to one log level, and for *P. stuartii* remained on the initial log level (0 h) throughout storage.

### 2.3. Experiment 3: Comparison of Bacterial Growth in Semen Diluted with Two Different Types of Antibiotic-Free Extenders

**Figure 3:**
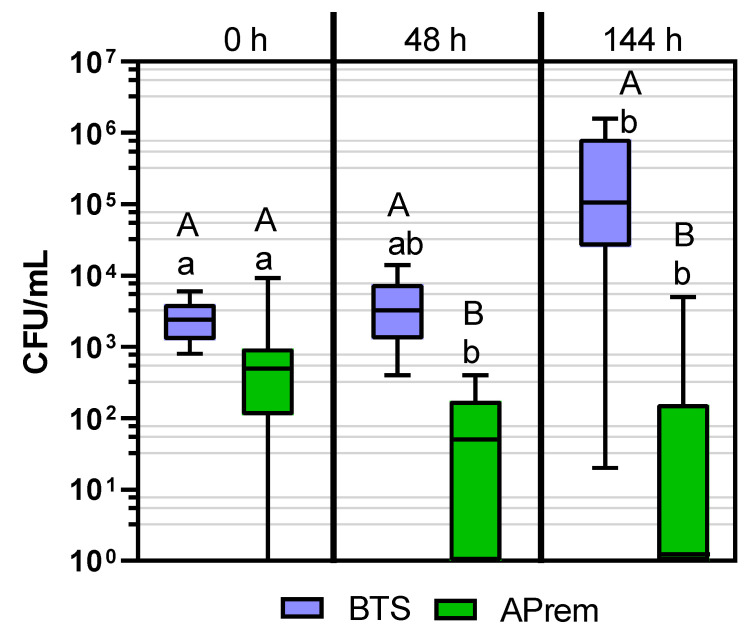
Bacterial counts (CFU/mL) in boar semen samples extended with antibiotic-free semen extenders Beltsville Thawing Solution (BTS) or Androstar Premium (APrem). Semen samples were inoculated with one of the four bacterial strains *P. stuartii*, *Ps. aer.*, *E. coli* and *Staph.* spp. and then stored for up to 144 h at 17 °C. Data are presented as box whisker plots (*n* = 12, Experiment 3). Data for each strain are shown in Supplementary Figure S1. A, B: Different capital letters indicate differences between extenders within storage time point (*p* < 0.05). a, b: Different lowercase letters indicate differences between storage time points within extender (*p* < 0.05).

The semen extender and the storage duration had a significant effect on bacterial counts (*p* < 0.05). The results are presented in Figure 3. Semen extended in Androstar Premium showed lower bacterial counts at 48 h and 144 h of storage compared to semen extended in BTS. After 144 h of storage, bacterial counts in Androstar Premium semen samples decreased to 4.6 × 10^2^ ± 1.4 × 10^3^ CFU/mL, whereas bacterial counts in BTS semen samples increased to 4.0 × 10^5^ ± 5.0 × 10^5^ CFU/mL. In semen samples extended in Androstar Premium and spiked with *E. coli* or *Staph.* spp., bacterial counts were reduced to <10^1^ CU/mL after 48 h and 144 h of storage at 17 °C; there was no increase in bacterial counts during 144 h in any of the spiked sample types (Supplementary Figure S1). In contrast, in semen samples extended in BTS, bacterial counts increased by one to two log levels in all four sample types.

### 2.4. Experiment 4a: Comparison of Bacterial Growth in Semen Diluted with Two Different Types of Extenders Containing Different Concentrations of Gentamicin

**Figure 4:**
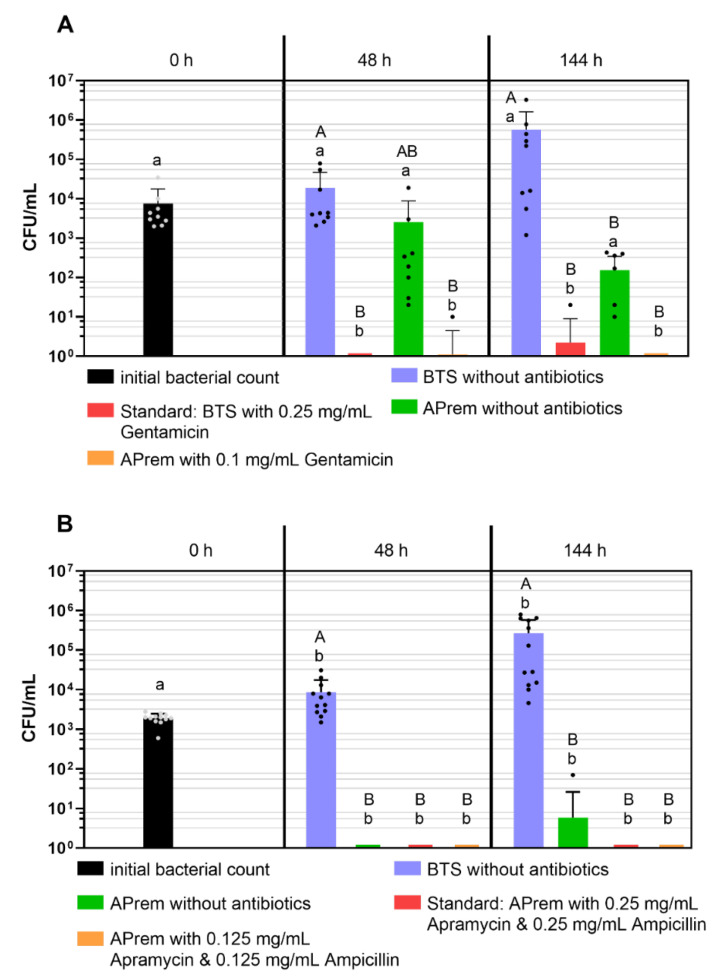
Bacterial counts (CFU/mL) in boar semen samples extended with Beltsville Thawing Solution (BTS) or Androstar Premium (APrem) containing different concentrations of antibiotics. Semen extenders were inoculated with a mixture of four bacterial strains (*P. stuartii*, *Ps. aer.*, *E. coli* and *Staph.* spp.) and then stored for up to 144 h at 17 °C. (**A**) the antibiotic gentamicin sulfate (*n* = 9, Experiment 4a). (**B**) antibiotic combination of apramycin sulfate and ampicillin (*n* = 9, Experiment 4b). All data are means and SD. A, B: Different capital letters indicate differences between extenders within storage time point (*p* < 0.05). a, b: Different lowercase letters indicate differences between storage time points within extender (*p* < 0.05).

The semen extender and the storage duration had a significant effect on bacterial counts (*p* < 0.05). The results are presented in Figure 4A. After 144 h storage at 17 °C in antibiotic-free semen samples, bacterial counts in Androstar Premium semen samples decreased to 1.5 × 10^2^ ± 1.8 × 10^2^ CFU/mL, whereas bacterial counts in BTS semen samples increased to 5.6 × 10^5^ ± 9.9 × 10^5^ CFU/mL. At 48 h and 144 h of storage, bacterial counts were <10^1^ CU/mL in semen samples in Androstar Premium with reduced (0.1 mg/mL) gentamicin and in BTS with the standard concentration of gentamicin (*p* > 0.05).

### 2.5. Experiment 4b: Comparison of Bacterial Growth in Semen Diluted with Androstar Premium Containing Different Concentrations of Apramycin and Ampicillin

The semen extender and the storage duration had a significant effect on bacterial counts (*p* < 0.05). The results are presented in Figure 4B. After 144 h of storage at 17 °C, bacterial counts in antibiotic-free BTS semen samples increased to 2.7 × 10^5^ ± 3.1 × 10^5^ CFU/mL (positive control), and in Androstar Premium decreased to <10^2^ CFU/mL. After 48 h and 144 h of storage, bacterial counts were <10^1^ CU/mL in semen samples in Androstar Premium samples both with reduced and with the standard concentration of apramycin and ampicillin (*p* > 0.05).

Sperm kinematic traits and sperm viability remained on a high level during 144 h of storage in all extender groups and fulfilled minimal requirements for use in artificial insemination (Table 1). After 144 h of storage, the total and progressive sperm motility in antibiotic-free Androstar Premium samples were significantly higher compared to Androstar Premium samples with the standard concentration of the antibiotics apramycin and ampicillin (*p* < 0.05).

## 3. Discussion

The present study confirms the two hypotheses; firstly, that antibiotic-free boar semen extenders display different antimicrobial capacities and secondly, that extenders with a higher bacteriostatic effect allow a reduction in currently used standard concentrations of antibiotics.

Typically, commercial boar semen extenders contain energy substrates, biological buffers, chelators and various electrolytes to preserve the viability of spermatozoa for several days in a liquid state. Based on these similarities to culture media, it has been suggested that semen extenders in the absence of antibiotics would favour bacterial growth similar to culture broth [23,24]. In the present study, we demonstrated that the BTS medium, one of the most common semen extenders, acts bacteriostatically to four typical commensal, mesophilic bacteria strains isolated from boar semen samples when compared to nutrient broth. The three Gram-negative bacterial strains used here represent the majority of the bacterial genera identified in boar ejaculates, which predominantly belong to the *Enterobacterales* and also the *Pseudomonadaceae* family [25,26,27,28]. The fourth test bacteria strain was *Staph.* spp., described as typical Gram-positive bacteria in raw boar semen in the cited studies. Obviously, the lack of ingredients from culture media in conjunction with a suboptimal storage temperature prior to aerobic culture at 37 °C inhibits bacterial growth in the semen extender. 

Our results showed that the bacteriostatic capacity differed between different types of semen extenders. One could suggest that so-called long-term extenders designed to maintain sperm longevity for up to seven days would not only better preserve sperm viability but also favour bacterial survival and growth. Interestingly, we observed just the opposite: the Androstar Premium extender, providing long-term preservability and cold shock protection for sperm [16,17], displayed a higher antimicrobial capacity compared to the BTS semen extender in the absence of antibiotics. This was visualised by a 2 to 3.5 log level lower presence of the four bacterial test strains after 144 h in semen-free Androstar Premium samples, three of them (*E. coli*, *Ps. aer.*, *Staph.* spp.) being below 10^1^ CFU/mL. At this stage, the reason for the divergent effect of the Androstar Premium extender on spermatozoa and bacteria remains elusive. Antimicrobial side-effects of enriched extender media may apply, perhaps in synergism with EDTA, a basic component of the semen extender [20]. Such a synergism could induce antimicrobial activity with non-antibiotic active molecules [29], as recently described for the synergetic antimicrobial effect of EDTA with the aminopeptidase inhibitor bestatin in extended rabbit semen [30]. 

The presence of seminal plasma and sperm may alter the antimicrobial efficiency of semen extenders by adding a plethora of proteins, lipids and electrolytes which might act as a substrate for bacteria. On the other hand, components acting as antimicrobial defence were identified in boar seminal plasma, for example, zinc-ion depending proteins [31] or lysozyme [32]. For this reason, follow-up experiments were performed with semen diluted analogue to the preparation of semen dose for routine AI, which resulted in 4.4 to 7.3% (*v*/*v*) seminal plasma with a fixed sperm number of 2 × 10^9^ per dose. To ensure sufficiently high contamination rates reflecting a reported bacterial load of 10^3^ to 10^6^ CFU/mL in boar ejaculates [26,27,28], the freshly extended semen doses were spiked with a mixture of the four model bacteria strains. Essentially, the observations of the semen-free Experiments 1 and 2 were confirmed by showing a superior antimicrobial capacity in semen extended in antibiotic-free Androstar Premium compared to the BTS medium. It is to note that throughout the long-term storage period, the bacterial load in the Androstar Premium samples remained below 10^5^ CFU/mL and thus did not exceed thresholds of several commensal bacteria considered harmful for sperm [33,34,35,36]. Some authors identified sperm at bacterial ratios of 1:1 as being the limit before sperm agglutination, loss of motility and membrane disintegration become apparent [24,37], which would translate to approximately 2 × 10^7^ CFU/mL in a semen dose commonly used for pig insemination. In accordance with these reports, in the present study, sperm motility and viability were maintained at a high level in the absence of antibiotics during 144 h of storage at 17 °C. Conversely, progressive motility at 144 h was reduced in samples preserved long-term with the extender, containing the standard concentration of a combination of apramycin and ampicillin. Likewise, reduced sperm motility has been previously shown for gentamicin at a concentration of 1 mg/mL or greater for preserved stallion and ram semen [38,39]. The detrimental effect of antibiotics on sperm motility is dose-dependent and may be enhanced during long-time exposure to liquid preserved sperm. Hence, a reduction in antibiotics in semen extenders serves not only to reduce environmental pollution but may also be beneficial to maintain sperm longevity. 

Complete removal of antibiotics from semen extenders is not regarded as safe for semen storage at 17 °C, but a lower dosage than that currently used in routine appears to be possible. This was shown in the final experiment where we demonstrated that in the Androstar Premium extender, the amounts of gentamicin and of the ampicillin/apramycin combination can be reduced to 50% of their regular concentration without losing antimicrobial capacity. It is to note that this study focused on typical commensal bacterial contaminants from the boar’s origin identified in raw and extended semen samples. The risk of transmission of specific pathogens in sow herds causing diseases such as brucellosis, chlamydophilosis and leptospirosis is minimised by effective biosecurity protocols [40]. Clearly, any effort to reduce or replace antibiotics in semen extenders must be accompanied by well-organised hygiene measures in the semen collection area and in the laboratory. Appropriate guidelines with proven effectiveness have been established [25,27].

## 4. Materials and Methods

### 4.1. Chemicals and Media

All chemicals were of analytical grade and purchased from Sigma-Aldrich Productions GmbH (Steinheim, Germany), Thermo Fisher Scientific, Inc. (Waltham, MA, USA), Enzo Life Sciences GmbH (Lörrach, Germany), Carl Roth GmbH & Co. KG (Karlsruhe, Germany), Merck KGaA (Darmstadt, Germany), Oxoid Deutschland GmbH (Wesel, Germany) and Beckman Coulter GmbH (Krefeld, Germany). Antibiotic-free semen extenders BTS [20] consisting of 205 mM D-Glucose Monohydrat (≥99.5% purity), 20.4 mM Na_3_C_6_H_5_O_7_ Dihydrat (≥99% purity), 10.0 mM KCL (≥99.5% purity), 15 mM NaHCO_3_ (≥99.5% purity) 3.36 mM EDTA (≥97% purity) and Androstar Premium were obtained from Minitüb GmbH (Tiefenbach, Germany). All extender media were sterile filtered before use. Nutrient broth consisting of 85.5 mM NaCl, 39 mM NaOH in 1000 µL aqua dest. supplemented with 10 g peptone and 10 g meat extract was freshly made and autoclaved in the Institute for Microbiology of the University of Veterinary Medicine Hannover, Foundation, Hannover, Germany.

### 4.2. Microbiology

#### 4.2.1. Bacterial Count

The total bacterial cell count in all samples was determined from a 10-fold serial dilution prepared in PBS ranging from 10^−1^ to 10^−6^. A volume of 100 μL of each dilution was plated on Columbia agar with sheep blood (Oxoid Deutschland GmbH) and incubated for 24 h at 37 °C under aerobic conditions. Bacterial numbers were calculated and expressed as colony-forming units per millilitre (CFU/mL). 

#### 4.2.2. Bacterial Inoculation of Samples

Samples were spiked with pure or mixed strains of bacteria naturally occurring in boar ejaculates. *P. stuartii*, *Ps. aer.*, *E. coli* and *Staph.* spp. were isolated from native semen of the used boars. The isolates were stored at −80 °C. At 24 h before inoculation, the bacteria strains were cultured on Columbia agar with sheep blood (Oxoid Deutschland GmbH, Wesel, Germany) and incubated over night at 37 °C. Grown colonies were diluted in 2 mL of sterile antibiotic-free extender medium and bacterial concentration was checked with density photometer (SDM5, Minitüb GmbH, Tiefenbach, Germany). Extenders or extended semen samples were spiked with this bacterial solution to a final bacterial concentration of approximately 5 × 10^3^ CFU/mL. The initial bacterial load in extenders or semen samples was determined immediately after inoculation (0 h). All microbiology experiments of this study were performed in an approved microbiology laboratory by trained personnel. 

### 4.3. Spermatology

#### 4.3.1. Semen Collection and Processing

Ten sexually mature and fertile boars (one to 5.5. years of age) of different breeds (Pietrain, Landrace, Duroc/Pietrain and Large White), housed in individual pens and treated in accordance with the European Commission Directive for Pig Welfare in the Unit for Reproductive Medicine, University of Veterinary Medicine Hannover, Foundation were used for semen collection. At weekly intervals, entire ejaculates without the bulbourethral gland secretion were collected by trained technicians using the gloved hand method. All procedures were approved by the institutional Animal Welfare Committee of the University of Veterinary Medicine Hannover, Foundation. All ejaculates fulfilled the standards for semen use in artificial insemination. Semen was extended with pre-warmed (35 °C) commercial extender to 20 × 10^6^ sperm/mL at a final volume of 100 mL. Semen samples were kept at room temperature for 90 min and then stored for 144 h in the dark at +17 °C.

#### 4.3.2. Assessment of Sperm Motility

The computer-assisted semen analysis (CASA) AndroVision^®^ (Version 1.2, Minitüb GmbH) equipped with an automated microscope warming stage, a digital camera (acA2440–75uc, Basler AG, Ahrensburg, Germany) and a TV adapter (60-C 1” 1.0×, Carl Zeiss Microscopy GmbH, Jena, Germany) was used to measure sperm motility as described by Höfner et al. [41]. Briefly, aliquots of semen samples were incubated at 38 °C in a water bath for 30 min under air and before measurement using a 20 µL counting chamber (Leja Products B.V., Nieuw Vennep, The Netherlands). At least 400 spermatozoa per sample were recorded in four to five fields of view at 100 × (ocular 10×, objective 10×, camera adapter 1×) at a rate of 30 pictures per 0.5 s. Spermatozoa were recorded as “motile” when their curved-line velocity was higher than 24 µm/s and the amplitude of lateral head displacement was higher than 1 µm. Spermatozoa were recorded as “progressively motile” when their curved-line velocity was higher than 42 µm/s or the straight-line velocity was higher than 15 µm/s. 

#### 4.3.3. Assessment of Sperm Membrane Integrity

The integrity of sperm plasma membranes and acrosomes was assessed using the ‘Cyto Flex’ flow cytometer (Beckman Coulter GmbH, Krefeld, Germany) equipped with ‘CytExpert 2.3′ Software (Beckman Coulter GmbH, Krefeld, Germany) as previously described by Höfner et al. [41]. Briefly, aliquots of the semen sample were stained with propidium iodide (PI; final concentration 1.0 µg/mL), fluorescein conjugated peanut agglutinin (FITC-PNA; final concentration 0.6 µg/mL) and Hoechst 33342 (H; final concentration 0.45 µg/mL). Stained samples were incubated for 5 min at 38 °C under air. Thereafter, 10,000 events were analysed with a medium flow rate of 30–40 µL/min. The H 33342 stain was used to distinguish DNA-containing cells from dirt particles.

#### 4.3.4. Assessment of Sperm Agglutinations

Five µL of samples were examined under a phase contrast microscope (Carl Zeiss Microscopy GmbH) with 200-fold magnification (ocular 10×, objective 20×, phase 2). At least three fields of view were examined, and the degree of agglutination was estimated based on the following criteria: 0 = None to a few sperm are agglutinated, 1 = Less than 20% of sperm were agglutinated, 2 = 20 to 40% of sperm were agglutinated, 3 = 40 to 60% of sperm were agglutinated, 4 = 60 to 80% of sperm were agglutinated and 5 = 80 to 100% of sperm were agglutinated. 

### 4.4. Experimental Design

#### 4.4.1. Experiment 1: Comparison of Bacterial Growth in Nutrient Broth and Antibiotic-Free BTS Extender

Nutrient broth and BTS were inoculated as described in Section 4.2.2 with pure strains of bacteria (*Staph.* spp., *E. coli*, *Ps. aer.* and *P. stuartii*). All samples were stored at 17 °C for up to 48 h. Immediately after inoculation (0 h) and after 48 h of storage, colony counts were determined for all samples as described in Section 4.2.1.

#### 4.4.2. Experiment 2: Comparison of Bacterial Growth in two different Types of Antibiotic-Free Extenders

Antibiotic-free semen extenders BTS and Androstar^®^ Premium were inoculated as described in Section 4.2.2 with pure strains of bacteria (*Staph.* spp., *E. coli*, *Ps. aer.* and *P. stuartii*). All samples were stored at 17 °C for up to 144 h. Immediately after inoculation (0 h), after 48 h and 144 h of storage, colony counts were determined for all samples as described in Section 4.2.1.

#### 4.4.3. Experiment 3: Comparison of Bacterial Growth in Semen Diluted with Two Different Types of Antibiotic-Free Extenders

Antibiotic-free semen extenders BTS and Androstar Premium were inoculated as described in Section 4.2.2 with one of the pure strains of bacteria: *Staph.* spp., *E. coli*, *Ps. aer.* and *P. stuartii*. The spiked extender media were used for diluting three different ejaculates. This resulted in 2 (extender) × 4 (bacterial strains) × 3 (boars) = 24 samples contaminated with an individual bacterial mixture consisting of the natural bacterial content from the ejaculate and the inoculated pure bacteria. All samples were stored at 17 °C for up to 144 h. Immediately after inoculation (0 h), after 48 h and 144 h of storage, colony counts were determined for all samples as described in Section 4.2.1.

#### 4.4.4. Experiment 4a: Comparison of Bacterial Growth in Semen Diluted with Two Different Types of Extenders Containing Different Concentrations of Gentamicin

The BTS and Androstar Premium extenders were used antibiotic-free and with gentamicin sulfate in two different concentrations: BTS with 0.25 mg/mL (standard) and Androstar Premium: 0.1 mg/mL. All samples were inoculated as described in Section 4.2.2 with the same mixture of bacteria consisting of *Staph.* spp., *E. coli*, *Ps. aer.* and *P. stuartii*. The spiked extender media were used for diluting nine different ejaculates from seven boars. This resulted in 4 (extenders) × 9 (boars) = 36 samples contaminated with the natural bacteria content from the ejaculate and the inoculated mixture of bacteria. All samples were stored at 17 °C for up to 144 h. Immediately after inoculation (0 h), after 48 h and 144 h of storage, colony counts were determined for all samples as described in Section 4.2.1. 

#### 4.4.5. Experiment 4b: Comparison of Bacterial Growth in Semen Diluted with Two Different Types of Extenders Containing Different Concentrations of Apramycin and Ampicillin

The BTS and Androstar Premium extenders were used antibiotic-free, and additionally the Androstar Premium was used with two different concentrations of a combination of apramycin sulfate and ampicillin: 0.25 mg/mL apramycin sulfate and 0.25 mg/mL ampicillin (standard), and 0.125 mg/mL apramycin sulfate and 0.125 mg/mL ampicillin. All samples were inoculated with the same mixture of bacteria consisting of *Staph.* spp., *E. coli*, *Ps. aer.* and *P. stuartii*. The spiked extender media were used for diluting 12 different ejaculates from six boars (two ejaculates per boar). This resulted in 4 (extenders) × 12 (boars) = 48 samples contaminated with the natural bacteria content from the ejaculate and the inoculated mixture of bacteria. All samples were stored at 17 °C for up to 144 h. Immediately after inoculation (0 h), after 48 h and 144 h of storage, colony counts were determined for all samples as described in Section 4.2.1. 

### 4.5. Statistical Analysis

Data were analysed using IBM SPSS Statistics Professional (SPSS Inc., IBM, Armonk, NY, USA). Data were checked for normal distribution with the Shapiro-Wilk Test. To address the repeated measurements, the Friedman Test (XLSX) was performed. Pairwise comparisons were performed with the Wilcoxon Test and corrected by Holm Bonferroni. Measurements were classified as significant when *p* < 0.05. All data are expressed as mean ± standard deviation (SD) or as box-whisker-plots including the median and 25th and 75th percentiles.

## 5. Conclusions

An intrinsic antimicrobial activity in antibiotic-free commercial semen extenders allows a reduction in the use of antibiotics in boar semen doses. Consideration of the specific antimicrobial capacity of semen extenders would greatly help to reduce the entry of antibiotics into the environment and could be beneficial for the quality of long-term stored sperm. This approach is economically feasible and can be instantly used until alternative antimicrobial strategies are adopted in artificial insemination of pigs.

## Figures and Tables

**Table 1:** Experiment 4b: Sperm quality in semen samples extended in Androstar Premium (APrem) with a standard concentration of 0.125 mg/mL apramycin and 0.125 mg/mL ampicillin (0.25 mg/mL AA) and reduced concentration of these antibiotics. Positive control: Semen extended in antibiotic-free Beltsville Thawing Solution (BTS). Data are means and SD (*n* = 9, Experiment 4b). Corresponding microbiology data are presented in Figure 4B.

Parameter	Extender	Antibiotics (Each)	48 h Storage	144 h Storage
Membrane intact (propidium iodide and FITC-PNA negative) sperm (%)	BTS	none	85.4 ± 7.8 ^a,A^	84.4 ± 7.7 ^a,B^
	APrem	none	86.7 ± 7.0 ^b,A^	85.8 ± 8.0 ^b,B^
	APrem	0.25 mg/mL AA	86.4 ± 7.6 ^b,A^	85.5 ± 7.7 ^b,B^
	APrem	0.125 mg/mL AA	86.5 ± 7.4 ^b,A^	85.3 ± 7.6 ^a,b,B^
Degree of agglutinations	BTS	none	1.1 ± 0.3 ^A^	2.1 ± 0.3 ^B^
	APrem	none	1.1 ± 0.3 ^A^	2.1 ± 0.3 ^B^
	APrem	0.25 mg/mL AA	1.1 ± 0.3 ^A^	2.1 ± 0.3 ^B^
	APrem	0.125 mg/mL AA	1.1 ± 0.3 ^A^	2.1 ± 0.3 ^B^
Total motility (%)	BTS	none	82.9 ± 8.7 ^a,A^	81.4 ± 9.7 ^a,A^
	APrem	none	85.3 ± 6.3 ^a,A^	84.6 ± 7.5 ^a,A^
	APrem	0.25 mg/mL AA	83.6 ± 8.1 ^a,A^	81.0 ± 10.2 ^a,B^
	APrem	0.125 mg/mL AA	83.2 ± 8.8 ^a,A^	84.1 ± 7.5 ^a,A^
Progressive motility (%)	BTS	none	76.9 ± 9.1 ^a,A^	73.9 ± 9.2 ^ab,B^
	APrem	none	79.5 ± 6.8 ^a,A^	78.2 ± 7.9 ^b,A^
	APrem	0.25 mg/mL AA	76.7 ± 8.2 ^a,A^	73.4 ± 9.8 ^a,B^
	APrem	0.125 mg/mL AA	76.6 ± 9.3 ^a,A^	76.7 ± 7.4 ^ab,A^
Straight line velocity (µm/s)	BTS	none	63.8 ± 7.4 ^a,A^	66.7 ± 9.4 ^a,A^
	APrem	none	73.9 ± 8.7 ^a,b,A^	73.7 ± 8.1 ^a,A^
	APrem	0.25 mg/mL AA	77.2 ± 11.6 ^b,A^	73.1 ± 7.6 ^a,A^
	APrem	0.125 mg/mL AA	77.3 ± 9.1 ^b,A^	73.2 ± 8.7 ^a,A^
Linearity	BTS	none	0.41 ± 0.09 ^A^	0.36 ± 0.05 ^a,A^
	APrem	none	0.38 ± 0.07 ^A^	0.35 ± 0.06 ^a,A^
	APrem	0.25 mg/mL AA	0.38 ± 0.08 ^A^	0.34 ± 0.05 ^a,A^
	APrem	0.125 mg/mL AA	0.39 ± 0.08 ^A^	0.34 ± 0.05 ^a,A^
Amplitude of lateralhead displacement (µm)	BTS	none	1.46 ± 0.47 ^a,A^	1.63 ± 0.29 ^a,A^
	APrem	none	1.73 ± 0.35 ^b,A^	1.91 ± 0.25 ^b,B^
	APrem	0.25 mg/mL AA	1.73 ± 0.31 ^b,A^	1.91 ± 0.23 ^b,A^
	APrem	0.125 mg/mL AA	1.75 ± 0.35 ^b,A^	1.94 ± 0.29 ^b,B^
BCF (Hz)	BTS	none	30.0 ± 4.4 ^a,A^	26.7 ± 2.2 ^B^
	APrem	none	28.4 ± 4.1 ^a,b,A^	24.9 ± 2.6 ^B^
	APrem	0.25 mg/mL AA	27.6 ± 3.1 ^b,A^	25.1 ± 2.5 ^B^
	APrem	0.125 mg/mL AA	27.5 ± 3.6 ^a,b,A^	25.4 ± 2.8 ^A^

^a,b^ Different lowercase letters indicate differences between extender within storage time point (*p* < 0.05). ^A,B^ Different capital letters indicate differences between storage time points within extender (*p* < 0.05)

## Data Availability

The data presented in this study are available on request from the corresponding author.

## Supplementary Materials

The following are available online at https://www.mdpi.com/article/10.3390/antibiotics10111319/s1, Figure S1: Bacterial counts (CFU/mL) in boar semen samples extended with antibiotic-free semen extenders Beltsville Thawing Solution (BTS) or Androstar Premium (APrem).
