# Supplementary material for: Antimicrobially Active Semen Extenders Allow the Reduction of Antibiotic Use in Pig Insemination
Source: Antibiotics (Basel). 2021 Oct 29;10(11):1319. doi: 10.3390/antibiotics10111319 (PMC8615084; doi:10.3390/antibiotics10111319)
Supplement: Supplementary file 1 [file antibiotics-10-01319-s001.zip › antibiotics-1413340-supplementary.pdf]

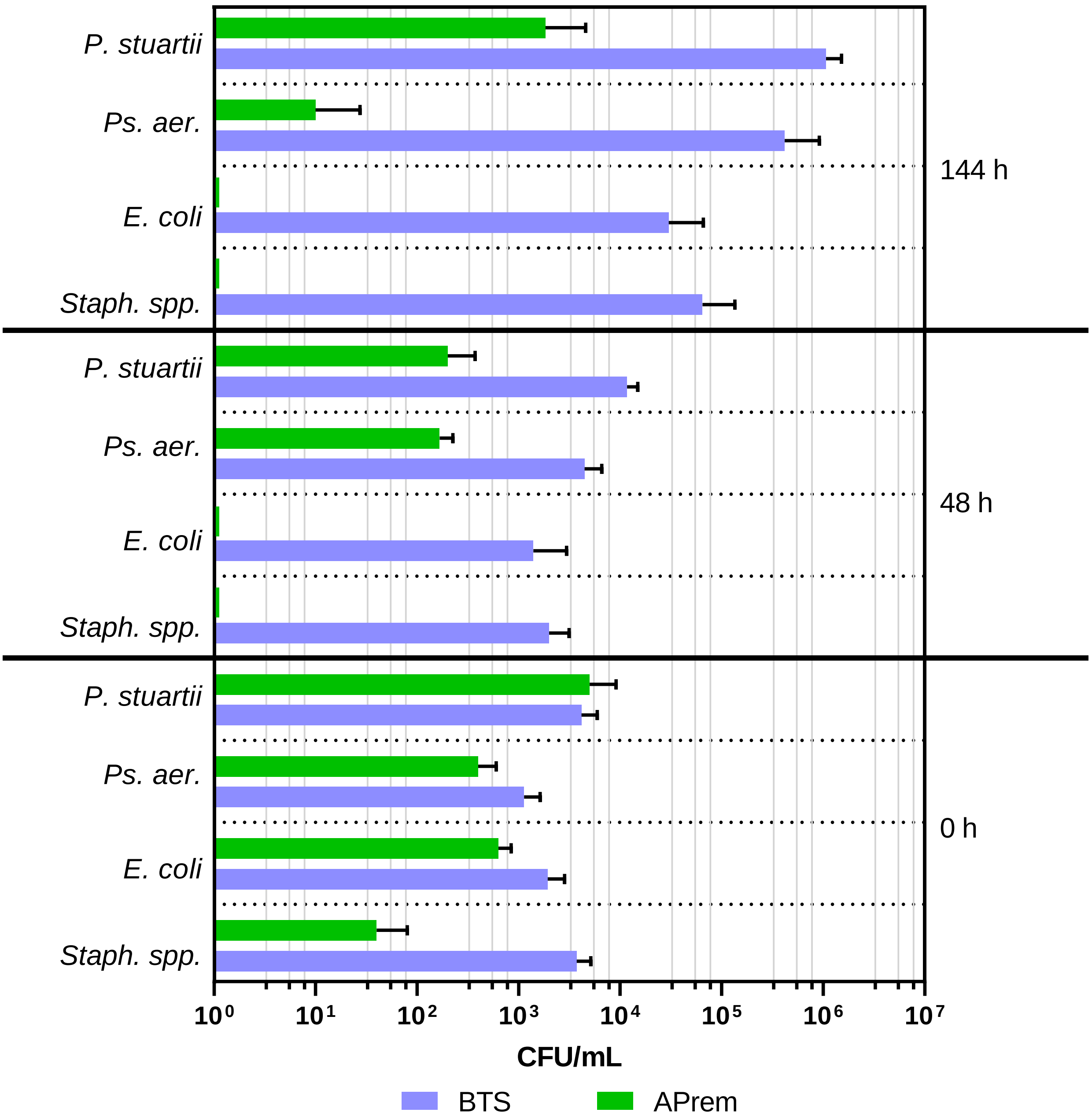

Supplementary Figure S1. Bacterial counts (CFU/mL) in boar semen samples extended with antibiotic-free semen extenders Beltsville Thawing Solution (BTS) or Androstar Premium (APrem). Semen samples were inoculated (0 h) with one of the four bacterial strains: *Staphylococcus species* (*Staph. spp.*), *Escherichia coli (E. coli)*, *Pseudomonas aeruginosa* (*Ps. aer*), *Providencia stuartii (P. stuartii)* and then stored for up to 144 h at 17 °C. Data are presented s means and standard deviation (n = 3 boars, Experiment 3).
